# Problematic Mobile Phone Use by Hong Kong Adolescents

**DOI:** 10.3389/fpsyg.2020.551804

**Published:** 2020-12-15

**Authors:** Joseph Wu, Aaron C. K. Siu

**Affiliations:** ^1^Department of Social and Behavioural Sciences, City University of Hong Kong, Kowloon, Hong Kong; ^2^Faculty of Education, The University of Hong Kong, Pok Fu Lam, Hong Kong

**Keywords:** problematic mobile phone use, Hong Kong adolescents, prevalence, risk factor, protective factor

## Abstract

**Background:**

Recently there have been growing concerns about problematic mobile phone use by adolescent populations. This study aimed to address this concern through a study of severity and correlates of problematic mobile phone use with a sample of Hong Kong adolescents.

**Methods:**

Data were collected from a sample of adolescents from three local secondary schools (ranging from high to low academic achievement levels) using a measuring scale (PCPU-Q, Yen et al., 2009) designated for Chinese adolescents. Participants were allocated into groups of “problematic users” and “non-problematic users” based on the number of occurrence of symptoms due to excessive and maladaptive use of mobile phone and possible functional impairments caused by problematic mobile phone use. A group of “at-risk users” was identified. A sample-based examination on distribution of these three groups of users was conducted via frequency counts and percentage calculation. A series of t-test were performed to make comparisons between “problematic” and “non-problematic” groups on selected personality and health related variable. Risk and protective factors were identified via correlational analysis and logistic regression analysis.

**Results:**

Under a more stringent cut-off criterion of four or more reported symptoms (out of seven) plus one or more reported functional impairments (out of five), 22.9% of the adolescents participating in this study could be classified as problematic mobile phone users. However, a more lenient criterion (only 4 or more reported symptoms without consideration of functional impairment) reported a substantially more severe prevalence rate (29.3%). A new group of “at-risk” adolescents (6.4%) was identified with such a discrepancy of prevalence rate. Gender difference, some risk and protective factors were also identified for developing this technology-related problem.

**Discussion and Conclusions:**

Adolescents who are vulnerable to suffer from this technology-related problem deserve more attention from helping professionals. Results of this study throw some insights on how to identify problematic mobile phone user applying a criterion-referenced approach. This study echoes a recent call for adopting a developmental perspective in understanding this problem and conducting research in this area. Anchored on present findings, effective interventions to tackle this rising problem among adolescents are suggested.

## Introduction

The mobile phone was once an expensive technological device that was affordable for only a few. Today, with substantial decreases in the cost of hardware procurement (the mobile phone and its accessories) and network connection (probably due to keen competition among service providers), the mobile phone has become almost an indispensable belonging for many people. In Hong Kong, a recent statistics from Office of the Communications Authority, the Government of the Hong Kong Special Administrative Region reported a mobile subscriber penetration rate of 275.9% as at March 2020 (Office of the Communication Authority, 2020). While a technology enriched environment can make lives easier, it may not lead to happiness for all (Oviedo-Trespalacios et al., 2016; Wang et al., 2017). Of all mobile phone user groups, adolescents are particularly keen to explore the possibilities of employing new technologies to enrich their lives (Csibi et al., 2019). Such an inclination toward new technologies may be beneficial to this group of users as they will find it easier than some other user groups to adapt to changing technological environments (Moore et al., 2019). However, for certain adolescents (e.g., those with low self-control), an over-exposure to technology may lead to certain kinds of behavioral addiction (Tang et al., 2015; Kim et al., 2016). Recently, there has been a rising concern about how an over-dependency on the use of the mobile phone may lead to deterioration of some adolescents’ psychological wellbeing (Sohn et al., 2019b). Regarding this concern, how to pull out individuals who are suffering (or will suffer) from this technology-related problem is pivotal for purposes of prevention and intervention (Shen and Su, 2019). This study aimed to address this concern from two perspectives, the prevalence of Hong Kong adolescents identified as problematic mobile phone users using a measuring instrument developed with a Chinese population, and the characteristics of this group of users.

### Prevalence of Problematic Adolescent Mobile Phone Users and Identification of an “At Risk” Group

How many adolescents can be identified as “problematic mobile phone users”? This is a critical question in this day and age. For most of the research in this area, researchers tend to adopt a loose definition on what entitles to be problematic use of mobile phone. In the present study, a widely cited definition of problematic use of mobile phone from Billieux (2012) was adopted to guide our investigation. According to Billieux (2012), problematic use of mobile phone can be regarded as “an inability to regulate one’s use of mobile phone, which eventually involves negative consequences in daily life” (p. 1). Systematic reviews on this topic suggest that excessive and maladaptive use and mental dysfunction caused by its use are two key elements being included in studies of this kind (Sohn et al., 2019b; Harris et al., 2020). In a recent systematic review on measuring scales of problematic mobile phone use conducted, Harris et al. (2020) found that most of these measuring scales have included items written on symptoms of increased and uncontrolled use of mobile phone as well as negative consequences arising from maladaptive use of mobile phone (e.g., poor academic performance, poor peer and familial relationships, compromised physical and/or psychological health, and financial problems). To further advance research in this area, they recommended a further standardization of these existing measuring instruments. Their critique on “excessive publication of seemingly redundant assessment tools” (Harris et al., 2020, p. 1) echo findings from another review that aimed to search for unique features of additive use of mobile phone (Yu and Sussman, 2020). After a review of 108 articles published between 2017 and 2019, Yu and Sussman (2020) asked for a more unified theory on mobile phone addiction for better identification of distinct additive traits of mobile phone use.

Arising from this lack of censuses on an exact definition of problematic mobile phone use, estimates of prevalence among adolescents in Hong Kong suffering from problematic use of mobile phone vary widely, from a low rate of around 3% (Leung, 2017) to a high rate of near 30% (Leung, 2008). A number of studies reported rates somehow between these two figures (e.g., Yen et al., 2009; Martinotti et al., 2011; Lopez-Fernandez et al., 2014). Without an accurate estimation of its prevalent rate, researchers and practitioners are difficult to mobilize existing resources to design effective intervention programs and efficient prevention plans to alleviate its negative impacts on their respective communities.

In a study with a sample of 1,529 British secondary school pupils aged between 11 and 18 years (Lopez-Fernandez et al., 2014), a prevalence rate of 10% was reported using a measuring instrument (the Mobile Phone Problematic Use Scale, Bianchi and Phillips, 2005) that was adapted from one designed for Spanish adults. In a recent study, de-Sola et al. (2017) conducted a psychometric analysis of the Mobile Phone Problematic Use Scale with a sample of 1,126 Spanish adults aged from 16 to 65 years. Up to 20.5% of respondents were classified as “problematic users.” Despite the fact that, in both of these studies, the Mobile Phone Problematic Use Scale had been adapted as a measure of mobile phone dependency, differences in the participants’ characteristics can partly explain this discrepancy. As well, the 4-year time gap between the studies may also have been a factor contributing to the inconsistencies.

A number of studies of adolescent populations show the need to pay more attention to the prevalence of problematic mobile phone use. For instance, a study with a large sample (*N* = 10,191) of adolescent students in Southern Taiwan identified 16.7% of respondents as problematic mobile phone users (Yen et al., 2009). Other studies of problematic mobile phone use reported a great variation on the prevalence rate ranging from 10% (British adolescents, Lopez-Fernandez et al., 2014) to 24.8% (Korean adolescents, Kwon et al., 2013).

Investigation into the reported details of these studies suggests that this inconsistency might be due to a lack of commonly agreed definitions of “problematic mobile phone use” (Yu and Sussman, 2020), the use of different measuring instruments (Harris et al., 2020), or the investigation of samples from different populations. Due to these concerns, we have examined various measuring instruments that was published at the time of implementation of this study (e.g., SAS-SV developed with Korean adolescents, Kwon et al., 2013; MPDS developed with Japanese university students, Toda et al., 2006; PCPU-Q developed with Chinese adolescents, Yen et al., 2009). After a careful consideration of age matching and cultural relevance, the Problematic Cellular Phone Use Questionnaire (PCPU-Q; Yen et al., 2009) was chosen as our key measuring instrument for assessment of problematic mobile phone use of Hong Kong adolescents. This is a 12-item self-report measure developed to measure problematic use of mobile phone of Chinese adolescent and has been validated with a large sample (*N* = 10,191) of school adolescents in Southern Taiwan. The first seven items assess the occurrence of symptoms due to excessive and maladaptive use of mobile phones, and the latter five items assess possible functional impairments caused by problematic mobile phone use. All items are written in traditional Chinese which is also the daily language used by Hong Kong adolescents in their school work. The participants were asked to choose “Yes” or “No” for each of the 12 items based on their phone usage during the month prior to the study. This dichotomous response format is suitable for adolescents with limited literary in reading survey items. Yen et al. (2009) recommended an optimal cut-off point of 4 or more symptoms (regardless of reporting any functional impairment or not) for the identification of problematic mobile phone use. A later study using the same measuring instrument (i.e., the PCPU-Q) reported a more stringent criterion (Wang et al., 2014). Specifically, the latter (more stringent) criterion required reporting of at least one functional impairment (out of 5) in addition to 4 or more endorsed symptoms (out of 7). The two separate cut-off criteria using the same measuring instrument pose a question to users of this scale: which cut-off criterion should be adopted? What are the differences between them?

An essential difference between the two criteria is the inclusion and exclusion of functional impairments arising from maladaptive use of mobile phones as a necessary indicator of “problematic use.” It echoes a recent discussion on whether mobile phone addiction should (or should not) be accepted as a real form of technological additions (Panova and Carbonell, 2018) and, if yes, how to identify this disorder along a continuum of addictive behaviors (Yu and Sussman, 2020). In the present study, instead of adopting a dichotomous view of cutting users into “problematic” versus “non-problematic” groups which are prevailing in studies of this kind, we apply a more developmental view for identification and classification of problematic mobile phone use. Specifically, it is proposed that a new group of mobile phone users should be located between normal/adaptive and problematic use of mobile phone. This newly proposed group is characterized by mild occurrence of symptoms due to excessive and maladaptive use of mobile phones but these symptoms has not yet been severe enough to cause any of the functional impairments identified in extant literature. Our proposal of adding an “at-risk” group between the normal and problematic mobile phone users is in line with the pathway model of problematic mobile phone use (Billieux et al., 2015) in this area of research. In the pathway model, addictive use of mobile phone is postulated as a psychological process that will take time to develop. By making elaboration on this model, we propose to add a new group of mobile phone users labeled as “at risk user” between the “non-problematic” and “problematic” groups. In the present study, percentage of this “at-risk” group is estimated with a sample of Hong Kong school adolescents using scores of PMPU-Q.

### Correlates of Problematic Mobile Phone Use Among Adolescents

Compared with other age groups, adolescents seem to be more vulnerable to this evolving type of technology addiction (Sharma et al., 2017). Before anything can be done to tackle the problem of increasing numbers of adolescents being identified as sufferers of maladaptive use of mobile phones, it is necessary to gain a better understanding of who these users are. Gender difference is one of the most frequently examined topics in this area of study (Sohn et al., 2019b). Earlier studies of internet addiction have suggested strongly that more males than females are addicted to internet use (Ha and Hwang, 2014). A possible explanation on it is that males may have tended to be more skillful in the use of technology in the days when it was less “user-friendly.” This gender difference has been reversed, however, in recent studies of problematic mobile phone use, with higher prevalences of addiction reported in females now. An inclination toward overuse of some easy-to-manage and free of charge mobile phone Apps for communication and social networking might be a reason for this reversal (Lopez-Fernandez et al., 2017). Though this kind of explanation sounds reasonable to explain the observed direction of gender differences, further evidence to confirm existence of this difference is still in place for data collected with different measuring instruments and populations.

Besides gender differences, some self-related personality constructs have been examined in this area of study. One of these, self-control, has drawn much attention in recent years (e.g., Özdemir et al., 2014; Tang et al., 2015; Kim et al., 2016; Han et al., 2017). Previous studies of self-control with behavioral addiction suggested that lack of self-control would be a risk factor of substance (e.g., drug) and non-substance (e.g., internet) addictions. Earlier research on problematic mobile phone use often adopted an atheoretical approach due to its newest in the field. With more and more research conducting on this topic, borrowing the conceptualization of non-substance addiction and its corresponding assessment criteria as written in DSM 5 (American Psychiatric Association, 2013) into research of problematic mobile phone use is a way to provide a theoretical foundation for studies in this area. Due to its exploratory nature, this study made an attempt to examine whether lack of self-control would act as a risk factor for a manifestation of non-substance addiction such as problematic use of mobile phone. Given the availability of huge amounts of information in cyberspace and multiple functions of mobile phones today, adolescents are attracted easily toward over-use of their phones to satisfy various needs in their lives. As an uncontrolled use is also an important symptom of problematic mobile phone use, it further motivates the inclusion of self-control into the list of risk factors in this study. With respect to the target population of this study, previous research with adolescent populations has provided some evidence about the importance of lack of self-control in predicting deviant behaviors in this population (e.g., Chui and Chan, 2015).

Another self-related construct that has received attention from researchers in this area of study is self-concept clarity (Sohn et al., 2019a; Kong et al., 2020; Wu et al., 2020). According to Erikson’s theory of psychosocial development (Erikson, 1968), a key developmental task of late adolescence is identity exploration. Activities like web-surfing and communication with friends via mobile phone are means to achieve this end. A recent study with a sample of 347 Korean university students reported an association of low self-identity with high mobile phone addiction (Kim, 2017). In a number of research on internet addiction (which shares a lot of characteristics with addiction to mobile phone use), self-identity confusion has shown to be a risk factor of this non-substance abuse (Hsieh et al., 2019). Along this line of thought, it has been speculated that adolescents with less articulated self-schema) tend to be more vulnerable to maladaptive uses of mobile phones. In research of the “*self*,” self-concept clarity is a construct tapping on how well an individual is holding an articulated, consistency, and stable view of the self. As such, we can anticipate that high self-concept clarity is a protective factor of problematic mobile phone use. Yet there is still a sparsity of research to either support or refute this speculation.

Within the context of Hong Kong, previous studies conducted by Leung (2008, 2017) have demonstrated a procedure of estimating prevalent rate of problematic mobile phone use through a norm-referencing approach. Present study proposed another way of making an estimation through a criterion-referencing approach. In terms of its novelty, an introduction of an “at risk” group of mobile phone user based on a developmental perspective and anchored on a pathway model of problematic mobile phone use (Billieux et al., 2015) will open a new area of research in this topic.

## Aims of the Study

In view of the rising trend of maladaptive mobile phone use in many technology-rich cities like Hong Kong, the present study aimed to:

(1) Estimate prevalent rate of problematic mobile phone use and “at risk” mobile phone use with a sample of Hong Kong adolescents; and
(2) Identify risk and protective factors associated with problematic mobile phone use by this sample of Hong Kong adolescents. Specifically, two personality constructs (self-control and self-concept clarity), six of purpose of mobile phone use (social networking, educational activities, instant messaging, enhancing productivity, and health management), and two measures of healthy lifestyle (sufficient sleeping time and regular physical exercise) were examined in this study. It was hypothesized that lack of self-control, heavy use of mobile phone for purposes of social networking or instant messaging, insufficient sleeping time, and irregular physical exercise were potential risk factors whereas high self-concept clarity would be a protective factor of problematic mobile phone use.

## Materials and Methods

### Procedure

In Hong Kong, schools are typically labeled as “Band 1,” “Band 2,” or “Band 3” according to the academic performances of their newly admitted secondary one student. In general, “Band 1” students perform better academically than “Band 2” students, who in turn perform better than their “Band 3” counterparts. Aside from academic performance, parents of “Band 1” students are typically richer and have access to more social resources than parents of “Band 2” students. Likewise, “Band 2” students are also socially and financially more advantaged than their “Band 3” counterparts. As majority of students from Band 1 secondary schools generally get a better academic performance than Band 2 and Band 3 students in the university entrance examination (i.e., the Hong Kong Diploma of Secondary Education), most students studying at Band 1 secondary school will aspire to continue their study upon completion of secondary education. As such, students from Band 1 secondary schools are more self-disciplined and more self-motivated in their academic studies. This variation in socioeconomic status and differential social expectation on students from different school banding may have an impact on characteristics and patterns of mobile phone usage. To generate a sample that would yield unbiased findings, it was considered necessary to collect data from each of these school types. Invitation letters were sent to school principals through the researchers’ social networks, and favorable responses were received from a number of schools. In view of the resources available for this study, three schools were selected for participating this study. Reasons of selecting these three schools included (a) a composition of one “Band 1,” one “Band 2,” and one “Band 3” schools could cover a full spectrum of students from different school banding and (b) locations of these schools from different geographical areas of the city could embrace a diversified sample characteristics for generation of unbiased findings. Informed consents were solicited from students and parents of the three schools selected to participated in the present study. A questionnaire composed of all measuring scales chosen and items developed for this study was administered by a trained research assistant to intact classes of students at the participating schools during a regular class time. Before administration of the survey, students were assured about their right to withdraw from the study at any point of time. Returned questionnares were examined for non-random pattern of missing responses and none of such kind of pattern could be identified. Questionnaires with any missing response(s) that made calculation of scale score(s) impossible were discarded in data analysis. As such, a final set of 575 questionnaires were retained for subsequent data analysis.

The study procedures were carried out in accordance with the guidelines laid down by the Ethical Review Committee of the City University of Hong Kong and approval was granted for the study by the Committee.

### Participants

A total of 588 completed questionnaire were returned from the three participating schools. After checking for missing responses in these returned questionnaire, 575 questionnaires were retained for data analysis, 273 were from girls (47.5%) and 296 (51.5%) were from boys; 6 respondents did not indicate their gender. Respondents were in either secondary 4 (*N* = 287) or secondary 5 (*N* = 278) grade levels at the time of data collection. Though the sample size of this study is not as large as study that collected data from a territory-wise sample, this sample of school adolescents shared most of the diverse characteristics of their peers at the same age.

### Instruments

#### Problematic Cellular Phone Use Questionnaire (PCPU-Q; Yen et al., 2009)

This is a 12-item self-report measure written in traditional Chinese and developed specifically for a population of Taiwanese adolescents. The first seven items assess the occurrence of symptoms due to excessive and maladaptive use of mobile phones, and the latter five items assess possible functional impairments caused by problematic mobile phone use. The participants were asked to choose “Yes” or “No” for each of the 12 items based on their phone usage during the month prior to the study. Reliability and validity of this measuring instrument was evident in a study reporting its development (Yen et al., 2009). Cronbach’s alpha for the seven symptoms items was reported as 0.85. Kappa coefficient of agreement of the items on the PCPU-Q ranged from 0.41 to 0.78 for a 2-week test–retest reliability and ranged from 0.26 to 0.44 between participants’ self-reports and their parents’reports. Participants classified as problematic users (i.e., reporting four or more symptoms in the first seven items) were more likely to suffer from depression as indicated by scores of a Chinese version of the Center for Epidemiological Studies’ Depression Scale (Chien and Cheng, 1985). The Cronbach’s α computed in this study was 0.73 for the first seven symtom items, 0.65 for the second five functional impairment items, and 0.79 for the total 12-item scale.

#### Self-Control Scale (SCS, Grasmick et al., 1993)

This is a 23-item scale measuring students’ abiities to have full control of their behaviors. There are some items that measure the personality traits of impulsivity (4 items), self-centeredness (4 items), risk-seeking (4 items), and ease of losing temper (4 items). There are also items that measure preference for engaging in simple tasks (rather than complicated ones, 4 items), and doing physical activities (rather than cognitive ones, 3 items). Responses were made on 4-point Likert scale, with anchors from “1 = strongly disagree” to “4 = strongly agree.” Scores from all 23 items were averaged. Higher scores indicated lower level self-control levels (i.e., higher levels of lack of self-control). A Chinese version of SCS (Weng and Chui, 2018) was adopted in this study and this version of the scale exhibited defensible psychometric properties with a Chinese student sample (*N* = 784). This Chinese SCS showed acceptable internal consistency reliability (Cronbach’s alpha = 0.78) and an underlying six-factor structure was demonstrated (Weng and Chui, 2018). The Cronbach’s α computed in this study was 0.85.

#### Self-Concept Clarity Scale (SCCS, Campbell et al., 1996)

This is a 12-item scale, of which 10 items should be reverse-keyed. Responses are made on a 5-point Likert scale, with anchors ranging from “1 = strongly disagree” to “5 = strongly agree.” A scale mean was computed by averaging the scores of all 12 items within the scale (with the reverse-keyed items recoded). The Chinese SCCS adopted in this study (Wu, 2012) were psychometrically sound. This version of scale showed good internal consistency (Cronbach’s alpha = 0.81), preservance of a one-factor structure in both exploratory and confirmatory factor analyses, and expected criterion validity with self-esteem and positive and negative affectivity (Wu, 2012). Higher scores indicate a higher clarity of the self. The Cronbach’s α computed in this study was 0.81.

#### Other Measures

The participants were asked to write down the percentage of time spent on each of the six given purposes of mobile phone use: (a) for social networking, (b) for educational activities; (c) for instant messaging; (d) for entertainment; (e) for enhancing productivity; and (f) for health management. The participants were also asked to report whether (“Yes” versus “No”) they spent sufficient time sleeping (7–8 h daily) and had adequate exercising time (at least twice weekly) during the previous month. Information about gender and the participant’s perceived academic performance (on a 5-point scale, from “1 = the worst” to “5 = the best”) was also collected.

### Statistical Analysis

All data analyses were done with SPSS version 26.0. To estimate the prevalence rate of problematic mobile phone use, frequency counts were done on responses to the first seven symptom items and the second five functional impairment items of the PCPU-Q. Identification of problematic mobile user was done using the cutoff criteria recommended by Yen et al. (2009; with 4 or more reported symptoms) and Wang et al. (2014; with 4 or more reported symptoms plus occurrence of any functional impairment), respectively. The percentage of adolescents who were regarded as “at risk” is estimated accordingly. To examine subgroup differences between problematic versus non-problematic mobile phone users, descriptive statistics of the study variables by this group membership were generated and comparisons were made across group membership. Risk and protective factors of problematic mobile phone use were assessed via logistic regression of group membership on the study variables.

## Results

### Prevalence of Problematic Mobile Phone Use

Table 1 shows the frequency counts of respondents who endorsed (i.e., checked “Yes”) the seven items measuring symptoms arising from and the five functional impairments caused by maladaptive use of mobile phones. To examine possible gender differences, these statistics were broken down further by sex. Ranking was done in descending order according to the total percentage of the full sample. From the table, it can be seen that the pattern was fairly consistent, in terms of ranking, across sexes for both the seven symptom items and the five functional impairment items. For the symptoms, at the top of the rank order was “tolerance: a marked increase in the frequency and duration of CPU,” followed by “withdrawal symptoms without CPU,” and “excessive time spent on CPU.” For the functional impairment, the item “poor academic performance” was ranked the highest, followed by “compromised physical or psychological function” and “poor relationship with friends or classmates.” The reported prevalence rate for girls was higher than for boys on all symptoms except one (the “continued heavy CPU”). However, only three of these sex differences reached a conventional level of statistical significance (i.e., *p* < 0.05, for the symptoms “tolerance: a marked increase in the frequency and duration of CPU,” “persistent desire and/or unsuccessful attempts to cut down or reduce CPU,” and “excessive time spent on CPU”), with a fairly weak effect size (ϕaround 0.10). In contrast, boys reported a higher prevalence rate on all except one functional impairment item (the exception being “poor academic performance”). Only one sex difference in functional impairment reached statistical significance (the item “problem in financial affairs” with *p* < .01), with a weak effect size (ϕ = 0.12). It should be noted that, after correction for possible inflation of type I error due to multiple comparisons was made via Bonferroni procedure (i.e., appliying adjusted alpha = 0.05/12 = 0.004), statistical sugnificance of all gender difference at item level of PCPU-Q vanished.

**TABLE 1:** Distribution of symptoms and functional impairments of problematic mobile phone use.

		Male (*n* = 296)^*a*^		Female (*n* = 273)		All (*n* = 575)			
		*N* (%)	*Rank*	*N (%)*	*Rank*	*N* (%)	*Rank*	*χ^2^(1)*	*ϕ*
** Symptoms of problematic mobile phone use**									
S1	Tolerance: a marked increase in the frequency and duration of CPU needed to achieve satisfaction	184 (62.2%)	1	193 (70.7%)	1	380(66.1%)	1	4.40*	0.09
S2	Withdrawal symptoms without CPU	139 (47.0%)	2	147 (53.8%)	2	291(50.6%)	2	2.57	0.07
S3	CPU for a period of time longer or more frequent than intended	87 (29.4%)	4	81 (29.7%)	5	169(29.4%)	4	0.00	0.00
S4	Persistent desire and/or unsuccessful attempts to cut down or reduce CPU	64 (21.6%)	6	32 (32.3%)	4	147(25.6%)	6	5.17	0.10
S5	Excessive time spent on CPU or excessive effort spent on activities necessary to obtain cellular phone	90 (30.4%)	3	107 (39.2%)	3	199(34.6%)	3	4.72*	0.09
S6	Giving up or reducing important social, academic, or recreational activities because of CPU	39 (13.2%)	7	39 (14.3%)	7	79 (13.7%)	7	0.14	0.02
S7	Continued heavy CPU despite knowledge of having a persistent or recurrent physical or psychological problem likely to have been caused or exacerbated by CPU	76 (25.7%)	5	69 (25.3%)	6	148(25.7%)	5	0.02	0.01
**Functional impairment caused by problematic mobile phone use**									
I1	Poor academic performance	98 (33.1%)	1	94 (34.4%)	1	195(33.9%)	1	0.09	0.01
1.I2	Poor relationship with friends or classmates	30 (10.1%)	4	20 (7.3%)	4	51 (8.9%)	4	1.43	0.05
I3	Poor relationship with family members	49 (16.6%)	3	32 (11.7%)	3	82 (14.3%)	3	2.77	0.7
I4	Compromised physical or psychological function	63 (21.3%)	2	47 (17.2%)	2	112(19.5%)	2	1.56	0.05
I5	Problems in financial affair	16 (5.4%)	5	3 (1.1%)	5	20 (3.5%)	5	8.20**	0.12

^a^The frequency counts are computed according to the number of respondents who endorsed the item of PCPU (i.e., ticked “Yes”). *p < 0.05, **p < 0.01.

### Identification of Problematic Mobile Phone User and Estimation of “At Risk” User

Table 2 shows the numbers of symptoms reported by the respondents and a cumulative count of these symptoms. When a more lenient criterion (Yen et al., 2009) was adopted (i.e., only a four-symptom criterion was used as a cut-off to identify problematic users), close to one-third of the respondents (29.3%) reported four or more symptoms with girls in the sample reported a higher prevalence rate (33.0%) than boys (26.9%) [χ^2^(1) = 2.52, *p* = 0.11]. Regarding the five functional impairments (see Table 3), only slightly more than half (55.6%) reported not having acquired any of these impairments, with less boys (in terms of percentage) reported that they were free from these functional impairments (53.9% versus 57.5%) [χ^2^(1) = 0.75, *p* = 0.39]. When a more stringent criterion was adopted as suggested by Wang et al. (2014) (i.e., use of 4 or more reported symptoms plus at least one reported functional impairment), slightly more than one-fifth (22.9%) could be classified as problematic users, with girls in the sample reported a higher prevalence rate than boys under this more stringent cut-off criterion (24.9% versus 20.9%) [χ^2^(1) = 1.17, *p* = 0.28]. It should note that the direction of gender difference observed in the current sample of respondents are in line with previous findings but this difference is not wide enough to reach a conventional level of statistical significance (i.e., a threshold of *p* < 0.05).

**TABLE 2:** Distribution of symptoms of problematic mobile phone use.

No of symptoms	Male (*n* = 294) *N* (%)	Female (*n* = 273) *N* (%)	All (*n* = 573) *N* (%)
0	56(19.0%)	33(12.1%)	90(15.7%)
1	65(22.1%)	65(23.8%)	131(22.9%)
2	69(23.5%)	52(19.0%)	122(21.3%)
3	25(8.5%)	33(12.1%)	60(10.5%)
4	33(11.2%)	32(11.7%)	65(11.3%)
5	25(8.5%)	35(12.8%)	60(10.5%)
6	9(3.1%)	14(5.1%)	23(4.0%)
7	12(4.1%)	9(3.3%)	22(3.8%)
Mean	2.29	2.63	2.46
Median	2	2	2
Mode	2	1	1

	**Male (*n* = 294)**		**Female (*n* = 273)**		**All (*n* = 573)**	
**Number of Symptoms reported**	***N***	**%**	***N***	**%**	***N***	**%**

7	12	4.1	9	3.	22	3.8
6 or more	31	7.1	23	8.4	45	7.9
5 or more	56	15.6	58	21.2	105	19.3
4 or more	89	26.9	90	33.0	170	29.3
3 or more	114	35.4	123	45.1	230	40.1
2 or more	183	58.8	175	64.1	352	61.4
1 or more	248	81.0	240	87.9	483	84.3

**TABLE 3:** Distribution of functional impairments caused by problematic mobile phone use.

No of Functional Impairment	Male (*n* = 294)	Female (*n* = 273)	All (*n* = 574)
	*N* (%)	*N* (%)	*N* (%)
0	159(53.9)	157(57.5)	319(55.6)
1	66(22.4)	66(24.2)	133(23.2)
2	35(11.9)	32(11.7)	68(11.8)
3	24(8.1)	9(3.3)	33(5.7)
4	7(2.4)	6(2.2)	13(2.3)
5	4(1.4)	3(1.1)	8(1.4)
Mean	0.87	0.72	0.80
Median	0	0	0
Mode	0	0	0

	**Male (*n* = 294)**		**Female (*n* = 273)**		**All (*n* = 574)**	
**Number of Functional Impairment reported**	***N***	**%**	***N***	**%**	***N***	**%**

5	4	1.4	3	1.1	8	1.4
4 or more	11	3.7	9	3.3	21	3.7
3 or more	35	11.9	18	6.6	54	9.4
2 or more	70	23.7	50	18.3	122	21.2
1 or more	136	46.1	116	425	255	44.4

In the present study only the group of respondents who satisfied Wang et al. (2014)’s criteria was categorized into “problematic mobile phone users.” There were 131 respondents (22.9%) in this group. There were 39 respondents (6.4%) who reported 4 or more symptoms but no functional impairments. Even this group of respondents could not be classified as “problematic mobile phone users” under the more stringent cut-off criterion, this was still a group of respondents who might easily develop into problematic mobile phone user. It can therefore be regarded as a group of “at risk” individuals who deserved attention from researchser and partitioners. There were 280 respondents (48.7%) who reported less than four symptoms with no functional impairment. Comparatively, this group of respondents could be regarded as “non-problematic users.” A potential contribution of this study is to demonstrate a practical way of identification of this group of mobile phone users using an existing measure of problematic mobile phone use with established psychometric integrity.

### Characteristics of Problematic Mobile Phone Use

To better understand the characteristics of problematic mobile users using the more stringent criteria as recommended by Wang et al. (2014), it was essential to compare this group of respondents with another group who could be classified as non-problematic mobile phone users. As noted by Knüppel and Hermsen (2010), splitting continuous populations into groups is a common practice in many fields of social sciences. Making decision on optimal number of groups to be formed should consider both efficiency gain and information loss. In research of problematic mobile phone use, dichotomize participants into groups of “problematic user” versus “non-problematic user” is a way to direct focus of researchers and resources of practitioners toward those who are in needs of attention, services and interventions. In the present study, a group of users between the “problematic” and “non-problematic” groups was added in. This group of users acquired only some characteristics of the “problematic” group (i.e., a sizeable number of reported symptoms but no functional impairment). However, given an ambivalent nature of this “at-risk” group as well as its novelty in the extant literature of research in this area, it was decided to exclude this group from the subsequent analysis of risk and protective factor to enhance interpretability and comparability of findings with previous studies. This practice could avoid incorporating confusing information that might come from the “at risk” group. As such, comparisons were only made between the “problematic” and “non-problematic users” in subsequent procedures of data anlysis (i.e., from Tables 4–6). The “at risk” group identified in this study was not included in the comparison because this group of adolescents would have an ambivalent characteristics associated with mobile phone use. Though allocating participants into three different groups anchored on scores of a continuous variable might render a risk of losing some important information on that variable, enhancement in interpretability of findings is a good reason to support such practice for this area of research (Sohn et al., 2019b; Harris et al., 2020; Yu and Sussman, 2020). After a careful consideration, it was decided that only the “problematic” group under the 4-or-more symptoms plus at least one reported functional impairment criteria and the “non-problematic” group with less-than-four symptoms plus no reported functional impairment were used for comparison, with an exclusion of the “risk-group” identified in this study. As shown in Table 4, compared with their counterparts, the “problematic users” were more likely to be female, lower in perceived academic performance, more frequent users of mobile phones for social networking and entertainment, and less frequent users of mobile phone for learning (and related activities) and self-management (e.g., health monitoring and prompting efficiency in daily work). Regarding personality, “problematic mobile phone users” reported lower self-concept clarity and self-control. With respect to lifestyle, they were less likely to have adequate sleep (7–8 h daily) or physical exercising time (at least doing physical exercise twice weekly).

**TABLE 4:** Descriptive statistics and group differences between problematic versus non-problematic user.

Variables		Problematic user (*N* = 131)	Non-problematic user (*N* = 280)	All (*N* = 411)		
						
	Response rang*e*	*M(SD)*	*M(SD)*	*M(SD)*	*t*	Cohen’s *d*
(1) Perceived academic achievement	1 (worst)–5 (best)	2.81 (0.91)	3.05 (0.91)	2.96 (0.91)	2.50*	0.27
(2) Self-control (lack of)	1 (low)–5 (high)	2.43 (0.37)	2.25 (0.42)	2.30 (0.41)	4.14***	0.46
(3) Self-concept clarity	1 (low)–5 (high)	2.75 (0.53)	3.12 (0.58)	3.00 (0.59)	5.95***	0.65
(4) Use for social networking (mainly for self-presentation)	0–100%	25.88 (15.08)	23.14 (14.52)	24.01 (14.73)	1.75	0.18
(5) Use for learning activities	0–100%	9.04 (8.24)	11.35 (10.50)	10.61 (9.88)	2.20*	0.24
(6) Use for instant messaging (mainly for interactive communication)	0–100%	25.90 (13.28)	26.00 (13.87)	25.97 (13.67)	0.07	0.01
(7) Use for entertainment	0–100%	32.36 (20.71)	26.96 (18.16)	28.68 (19.15)	2.67**	0.28
(8) Use for enhancing productivity	0–100%	3.97 (6.44)	4.2 (5.64)	4.13 (5.90)	0.37	0.04
(9) Use for health management	0–100%	3.14 (6.19)	3.46 (6.62)	3.36 (6.48)	0.46	0.05

	***Response option***	***N(%)***	***N(%)***	***N(%)***	***χ^2^(1)***	***ϕ***

(10) Adequate sleeping time	Yes/No	54 (41.5%)	156 (55.9%)	210 (51.3%)	7.34**	0.13
(11) Adequate physical exercise	Yes/No	60 (46.2%)	158 (56.8%)	218 (53.4%)	4.06*	0.10

*p < 0.05, **p < 0.01, ***p < 0.001.

**TABLE 5:** Intercorrelations of study variables in logistic regression analysis.

		1	2	3	4	5	6	7	8	9	10	11	12
(1)	Group membership												
(2)	Gender	0.03											
(3)	Perceived academic achievement	−0.12*	–0.01										
(4)	Self-control (lack of)	0.20***	–0.01	–0.06									
(5)	Self-concept clarity	−0.29***	–0.07	0.14**	−0.31***								
(6)	Use for social networking (for self-presentation)	0.09	0.18***	0.05	0.01	0.01							
(7)	Use for learning activities	−0.11*	0.00	0.05	−0.09*	0.08	−0.14**						
(8)	Use for instant messaging (for interactive communication)	–0.00	0.05	0.07	0.01	−0.08*	0.16***	−0.10*					
(9)	Use for entertainment	0.13**	−0.16***	0.02	0.03	–0.07	−0.24***	–0.06	−0.31***				
(10)	Use for enhancing productivity	–0.02	0.01	0.06	–0.05	0.09*	−0.09*	0.16***	–0.07	−0.10*			
(11)	Use for health management	–0.02	–0.02	–0.04	0.02	0.00	–0.04	0.11*	−0.08*	−0.09*	0.43***		
(12)	Sufficient sleeping time	−0.13**	–0.08	0.10*	–0.05	0.22***	–0.07	0.17***	–0.07	–0.02	0.01	0.02	
(13)	Regular physical exercise	−0.10*	−0.22***	–0.01	–0.04	0.13**	–0.02	0.08	–0.06	–0.07	0.09*	0.10*	0.15***

*p < 0.05, **p < 0.01, ***p < 0.001.

**TABLE 6:** Summary of logistic regression analysis for prediction of problematic phone use.

	Step 1			Step 2			Step 3			Step 4			Step 5		
Predictors	*B*	*SE B*	Exp*(B)*	*B*	*SE B*	Exp*(B)*	*B*	*SE B*	Exp*(B)*	*B*	*SE B*	Exp*(B)*	*B*	*SE B*	Exp*(B)*
(1) Gender	0.19	0.23	1.23	0.19	0.23	1.21	0.21	0.24	1.23	0.21	0.25	1.24	0.09	0.26	1.10
(2) Perceived academic achievement				–0.28	0.13	0.76*	–0.19	0.13	0.82	–0.24	0.14	0.79	–0.22	0.14	0.80
(3) Self-control (lack of)							0.78	0.33	2.19*	0.66	0.35	1.93	0.69	0.35	1.99
(4) Self-concept clarity							–0.97	0.23	0.38**	–1.03	0.24	0.36***	–0.96	0.24	0.39***
(5) Use for social networking (for self-presentation)										0.03	0.010	1.03**	0.03	0.01	1.03**
(6) Use for educational activities										–0.01	0.02	0.99	–0.01	0.02	0.99
(7) Use for instant messaging (for interactive communication)										0.01	0.011	1.01	0.01	0.01	1.01
(8) Use for entertainment										0.02	0.01	1.02**	0.02	0.01	1.02*
(9) Use for enhancing productivity										0.04	0.02	1.04	0.04	0.02	1.04
(10) Use for health management										–0.01	0.02	0.99	–0.01	0.02	0.99
(11) Sufficient sleeping time													–0.42	0.26	0.66
(12) Regular physical exercise													–0.28	0.26	0.75

*p < 0.05, **p < 0.01, ***p < 0.001. Model 1: Cox and Snell R^2^ = 0.00; Model χ^2^(1) = 0.70, p = 0.40. Model 2: Cox and Snell R^2^ = 0.02; Model χ^2^(2) = 5.85, p = 0.05; Change Cox and Snell R^2^ = 0.01; Change Model χ^2^(1) = 5.15, p = 0.02. Model 3: Cox and Snell R^2^ = 0.10; Model χ^2^(4) = 40.89, p < 0.001; Change Cox and Snell R^2^ = 0.08; Change Model χ^2^(2) = 35.04, p < 0.001. Model 4: Cox and Snell R^2^ = 0.14; Model χ^2^(10) = 57.20, p < 0.001; Change Cox and Snell R^2^ = 0.04; Change Model χ^2^(6) = 16.31, p = 0.01. Model 5: Cox and Snell R^2^ = 0.15; Model χ^2^(12) = 61.61, p < 0.001; Change Cox and Snell R^2^ = 0.01; Change Model χ^2^(2) = 4.41, p = 0.11.

### Risk and Protective Factors of Problematic Mobile Phone Use

Table 5 shows the correlations of group membership (problematic versus non-problematic users) with other variables in this study. As the direction and size of correlations of group membership with other variables echo the findings of subgroup analysis described in the previous section, they are not repeated here. These correlations were generally in a weak-to-moderate range and thus multicollinearity was probably not an issue in running regression analysis with these variables as predictors.

There were five steps in the specifications of the logistic regression model for identification of risk and protective factors associated with mobile phone use (see Table 6). In steps 1 and 2, Gender and Perceived Academic Achievement were entered. These two variables could serve as controls to better understand the effects of variables entered in subsequent steps. In step 3, the two personality variables (Self-control and Self-concept Clarity) were entered. In step 4, the six categories of purpose of mobile phone use were entered (social networking, learning activities, instant messaging, enhancing productivity, and health management). In the final step, two lifestyle variables were entered (sufficient sleeping time and regular physical exercising time). The results showed that variables in the first two steps of the regression (Gender and Perceived Academic Achievement) accounted for only a very small portion of the explained variance (around 2%) of the criterion variable (i.e., the Group Membership). The two personality variables in step 3 explained a further 8% of the variance of the criterion variable. The six mobile phone usage variables and two lifestyle variables accounted, respectively, for another 4 and 1% of the explained variance of the criterion variable. Altogether, the 12 predictors explained a total of 15% of the variance of the Group Membership.

At parameter level, estimates of regression coefficients (exponential beta) for six (Gender, Self-control, Use for Social Networking, Use for Instant Messaging, Use for Entertainment, and Use for Enhancing Productivity) out of 12 predicting variables in the final model (i.e., the regression model built in step 5) yielded a value larger than one, with two of these (Use for Social Networking and Use for Entertainment) reaching at least a conventional level of statistical significance (i.e., *p* < 0.05). For the remaining six predictors, estimates of the regression coefficient (exponential beta) had a value smaller than one, with one (Self-concept Clarity) reaching a conventional level of statistical significance.

## Discussion

There were two major goals in the present study. The first was to estimate prevalent rate of problematic mobile phone use and “at risk” mobile phone use with a sample of Hong Kong school adolescents. The second goal was to identify risk and protective factors of mobile phone use by this sample of Hong Kong adolescents.

Regarding the first goal, measured by the PCPU-Q (Yen et al., 2009) and with a cut-off criterion of reporting at least four symptoms (out of seven) and one functional impairment (out of five) suggested by Wang et al. (2014), the estimate based on responses from the current sample of Hong Kong school adolescents was 22.9%, with a higher rate for girls than for boys (24.9% versus 20.9%). This figure deserves attention from parents, teachers, counselors, researchers, and other personnel who care about the personal development of adolescents in this and future generations.

In the present study, a group of adolescents “at risk” in their mobile phone use was identified. This group of adolescents shared a characteristic of displaying considerable numbers of symptoms due to excessive and maladaptive use of mobile phones but its severity has not yet caused any functional impairments to their daily lives. This group represented 6.4% of respondents in the sample of this study. As with the development of other addictive behaviors (such as substance addiction), adolescents often follow a trajectory of starting from heavy use, then developing a habit of over and uncontrolled use, finally becoming addictive users of mobile phone. This echoes the view that a dichotomous categorization of mobile phone use (“problematic” versus “non-problematic”) might be an over-simplistic approach to understanding this problem (Panova and Carbonell, 2018; Yu and Sussman, 2020). A more developmental perspective is necessary to tackle this problem (Billieux et al., 2015). For example, the current findings have suggested a need to formulate appropriate interventions for “at risk” adolescents who already show symptoms of maladaptive use of mobile phones but not yet any functional impairments.

The above discussion leads to a further dialog about whether criterion-referencing or norm-referencing approaches should be used to classify the issue of “problematic mobile phone use.” For example, Leung (2017) adopted a criterion of two standard deviations above the sample mean as a cut-off and this yielded a prevalence rate estimate of 3.41% in Hong Kong school adolescents. The norm-referencing approach would cause the interpretation of the prevalence rate to become relative and ambiguous. Would scores above two standard deviations be interpreted as heavy or over-use of mobile phones? In contrast, the criterion-referencing approach could give a more concrete and interpretable meaning to the classification. For instance, when applying the 4-symptoms-plus-one-functional-impairment cut-off criterion of PCPU-Q, individuals classified as “problematic mobile phone user” could be regarded as those who have already reported addictive symptoms of maladaptive mobile phone use and are suffering from some functional impairment arising from its maladaptive use. In recent discussions of this issue, most researchers favor an adoption of the criterion-referencing approach (Sohn et al., 2019b; Harris et al., 2020; Yu and Sussman, 2020). With this consensue, a dialog on what are the necessary and sufficient conditions for identification of problematic mobile phone use is still on-going.

Regarding the second goal, both the subgroup and regression analysis have provided some weak evidence to support previous findings in a direction that girls were more vulnerable than boys to problematic use of mobile phones (Gezgin et al., 2018; Sohn et al., 2019b). Previous research on Internet addiction has consistently reported higher prevalence rates for males (Kuss et al., 2014). As already mentioned earlier in this paper, this may be explained by the idea that a decade ago it was more complex to connect to the Internet via computers, and that males may have able to do this better. However, given a small effect size of gender difference in this study, this speculative explanation warrants further examination.

In the final model of the logistic regression (step 5), even with the inclusion of a number of variables from different domains (demographic, academic, personality, purpose of mobile phone use, and healthy lifestyle) that previous research has shown to be related, both theoretically and empirically, to maladaptive mobile phone use, the explanatory power was still not very high (altogether accounting for a total of 15% of the variance). As there are so many personal, social, environmental, and cultural factors that can impact on one’s intentions and behaviors regarding mobile phone use, it is not surprising that the limited number of varables used in this study may not have been powerful enough to explain fully this very complicated problem. Even so, there are still some noteworthy findings from the series of regression analyses.

Among the four incremental changes in explained variance of the dependent variable (i.e., from step 1 to step 2, step 2 to step 3, step 3 to step 4, and step 4 to step 5), an addition of two personality variables (i.e., self-control and self-concept clarity) yielded the largest change (an increment of 8% explained variance). Within these two personality factors, lack of self-control was found to be a risk factor [with exp(*B*) > 1] whereas high self-concept clarity was shown to be a protective factor [with exp(*B*) < 1]. Many adolescents nowadays are motivated to use mobile phones excessively for activities that give them satisfaction in various life domains (e.g., knowledge acquisition, maintainance and extension of social networks, understanding of the self). If there are some environmental factors that may encourage unlimited satisfaction of these needs (provision of unlimited connection time under some subscription plans), adolescents with low self-control will be trapped into develop patterns of problematic use. The formulation of effective prevention and intervention strategies to alleviate the problem could focus on self-control training as informed by this study.

The extant literature provided two very different (and somehow contrasting) views on how self-concept clarity may relate to problematic mobile phone use. A recent study of the use of mobile phones for the purpose of self-presentation suggested that people will make frequently switching upward and downward social comparisons to evaluate themselves (Fardouly and Vartanian, 2015). These switching comparison processes can lead to a fragmentation of self-image and may do further harm to the development of integrated self-concepts. In contrast, those who found a negative relationship between these two constructs have argued for a beneficial use of mobile phones. Specifically, those with high self-concept clarity tend to search for information that will confirm their “good” selves. This kind of self-selection and self-affirmation process will further strengthen a stronger sense of the self. As such, use of the mobile phone as a tool of self-exploration will be beneficial in promoting psychological wellbeing (Fullwood et al., 2016). Data from this study support the perspective that high self-concept clarity can be a protective rather than a risk factor. Our study findings suggest that individuals who know clearly who they are will be more able than their counterparts with a confused sense of the self to make more beneficial use of mobile phone in their lives.

The six purposes of mobile phone use, together, accounted for an incremental total of 4% of the explained variance of problematic mobile phone use. At parameter level, four purposes of use (social networking, instant messaging, entertainment, and enhancing productivity) were identified as risk factors [with Exp (*B*) > 1] of problematic mobile phone use, with two of these regression coefficients (use for social networking and use for entertainment) reaching conventional levels of statistical significance. It is not surprising to see that many adolescents (especially boys) are losing self-control in the use of mobile phones when playing paid online games and making self-presentation through online social networking platforms.

The two aspects of healthy lifestyles (sufficient sleeping time and regular physical exercise) examined in this study were both found to be protective factors of mobile phone use for this sample of respondents. Though their predictive powers are not strong enough to arrive at any affirmative conclusion, directionality of this finding can still give a hint of a tip for alleviating the rising problem of maladaptive mobile phone use. As it is difficult and takes time to change one’s personality (like self-control and self-concept clarity), parents and practitioners (e.g., school counselors and social workers) can think about diverting adolescents’ energies and attention toward participating in physical activities (e.g., playing ball games) that can give them satisfaction and rewarding experiences.

There is also an observation that the magnitude of regression coefficient [exp(*B*)] of lack of self-control dropped slightly and became statistically non-significance (i.e., *p* > .05) with subsequent addition of six purposes of mobile phone use variables (in step 4) and two lifestyle variables (in step 5) into the regression model. This change of statistical significance could be attributed to possible effect of multicollinearity between exiting and newly added variables in the model. As a final remark, the role of lack of self-control as a risk factor and two healthy lifestyles (sufficient sleeping time and regular physical exercise) as protective factors need further examination in research of problematic mobile phone use.

There are several limitations that suggest caution when making interpretations of the findings of this study. First, a more rigorous evaluation of psychometric properties of all measuring instruments used in this study is necessary to better defend validity of findings from this study. Extensive use of reversed-keyed Likert type items as found in the Self-concept Clarity Scale need more attention in future research (Suárez-Álvarez et al., 2018; Vigil-Colet et al., 2020). Second, categorization of respondents into different groups anchored on scores of PCPU-Q and applying the group membership in subsequent correlational and regression analysis will render a risk of loosing some information conveyed by actual scores of the measuring instrument. Further study should revisit legitimacy of this practice. Third, like all self-report surveys, the participants may have tended to under-report the occurrence of symptoms and functional impairments. The reported prevalence rate might in fact be an under-estimate of the severity of the problem. Fourth, due to the limited resources available for this study, only the variables that have been shown previously to be related closely to problematic mobile phone use were included in this study. In future research, more variables at personal and contextual levels can be added to build up a comprehensive model of this issue. Fifth, due to the cross-sectional nature of the data, risk and protective factors identified in this study should be viewed as associative rather than predictive in nature. Further studies can employ more rigorious research designs (e.g., experimental design) or collect different types of data (e.g., longitudinal data, data from parents and teachers) to better examine possible causal relationships between the study variables.

## Conclusion

Findings from this study suggest that there are increasing number of adolescents suffering from problematic use of mobile phone. Adolescents who are vulnerable to suffer from this technology-related problems deserve more attention from helping professionals. Results of this study throw some insights on how to identify a group of adolescents who might be at risk of suffering From problematic mobile phone use. This study also demonstrated how to pull out this group of individuals using a measuring instrument with well-established psychometric integrity and applying a criterion-referencing approach. Practitioners (e.g., social workers and counsellors) should be more proactive to identify the at-risk individuals in their community and provide adequate services to them before they all turned into problematic users. Anchored on present findings, effective interventions to tackle this rising problem among adolescents are recommended. The intervention programs can focus on self-awareness training so that attendees may pick up a deeper understanding on who they are and what they need. Adolescents should also be taught to make a good control on how to use of mobile phone for the purposes of social networking and entertainment. For other risk and protective factors being examined in this study, further research are necessary to determine their roles in problematic use of mobile phone from different cultures and age groups.

## Data Availability Statement

The raw data supporting the conclusions of this article will be made available by the authors, without undue reservation, to any qualified researcher.

## Ethics Statement

The studies involving human participants were reviewed and approved by Ethical Review Committee of the City University of Hong Kong. Written informed consent to participate in this study was provided by the participants’ legal guardian/next of kin.

## Author Contributions

JW designed the study and compiled the questionnaire. AS collected data. JW and AS conducted the literature review and conducted the statistical analysis. JW wrote the first draft of the manuscript. Both authors contributed to and have approved the final manuscript.

## Conflict of Interest

The authors declare that the research was conducted in the absence of any commercial or financial relationships that could be construed as a potential conflict of interest.

## Abbreviations

CPU: Cellular Phone Use
PCPU-Q: Problematic Cellular Phone Use Questionnaire
SCS: Self-Control Scale
SCCS: Self-Concept Clarity Scale.
